# Somatosensory network functional connectivity differentiates clinical pain phenotypes in diabetic neuropathy

**DOI:** 10.1007/s00125-021-05416-4

**Published:** 2021-03-25

**Authors:** Kevin Teh, Iain D. Wilkinson, Francesca Heiberg-Gibbons, Mohammed Awadh, Alan Kelsall, Shillo Pallai, Gordon Sloan, Solomon Tesfaye, Dinesh Selvarajah

**Affiliations:** 1 Academic Department of Magnetic Resonance Imaging, University of Sheffield, Sheffield, UK; 2 Department of Oncology and Human Metabolism, University of Sheffield, Sheffield, UK; 3 Diabetes Research Department, Sheffield Teaching Hospitals NHS Foundation Trust, Sheffield, UK

**Keywords:** Diabetic neuropathy, Machine learning, MRI, Painful diabetic neuropathy, Resting-state functional MRI

## Abstract

**Aims/hypothesis:**

The aim of this work was to investigate whether different clinical pain phenotypes of diabetic polyneuropathy (DPN) are distinguished by functional connectivity at rest.

**Methods:**

This was an observational, cohort study of 43 individuals with painful DPN, divided into irritable (IR, *n* = 10) and non-irritable (NIR, *n* = 33) nociceptor phenotypes using the German Research Network of Neuropathic Pain quantitative sensory testing protocol. In-situ brain MRI included 3D T1-weighted anatomical and 6 min resting-state functional MRI scans. Subgroup differences in resting-state functional connectivity in brain regions involved with somatic (thalamus, primary somatosensory cortex, motor cortex) and non-somatic (insular and anterior cingulate cortices) pain processing were examined. Multidimensional reduction of MRI datasets was performed using a machine-learning approach to classify individuals into each clinical pain phenotype.

**Results:**

Individuals with the IR nociceptor phenotype had significantly greater thalamic–insular cortex (*p* false discovery rate [FDR] = 0.03) and reduced thalamus–somatosensory cortex functional connectivity (*p*-FDR = 0.03). We observed a double dissociation such that self-reported neuropathic pain score was more associated with greater thalamus–insular cortex functional connectivity (*r* = 0.41; *p* = 0.01) whereas more severe nerve function deficits were more related to lower thalamus–somatosensory cortex functional connectivity (*r* = −0.35; *p* = 0.03). Machine-learning group classification performance to identify individuals with the NIR nociceptor phenotype achieved an accuracy of 0.92 (95% CI 0.08) and sensitivity of 90%.

**Conclusions/interpretation:**

This study demonstrates differences in functional connectivity in nociceptive processing brain regions between IR and NIR phenotypes in painful DPN. We also establish proof of concept for the utility of multimodal MRI as a biomarker for painful DPN by using a machine-learning approach to classify individuals into sensory phenotypes.

**Graphical abstract:**

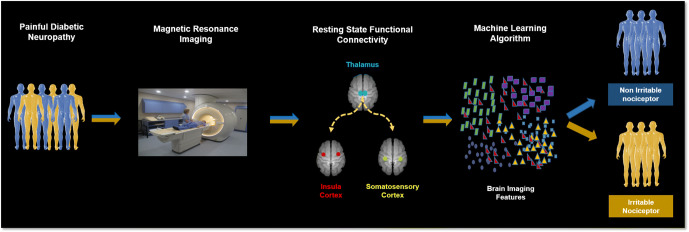

**Supplementary Information:**

The online version of this article (10.1007/s00125-021-05416-4) contains peer-reviewed but unedited supplementary material.

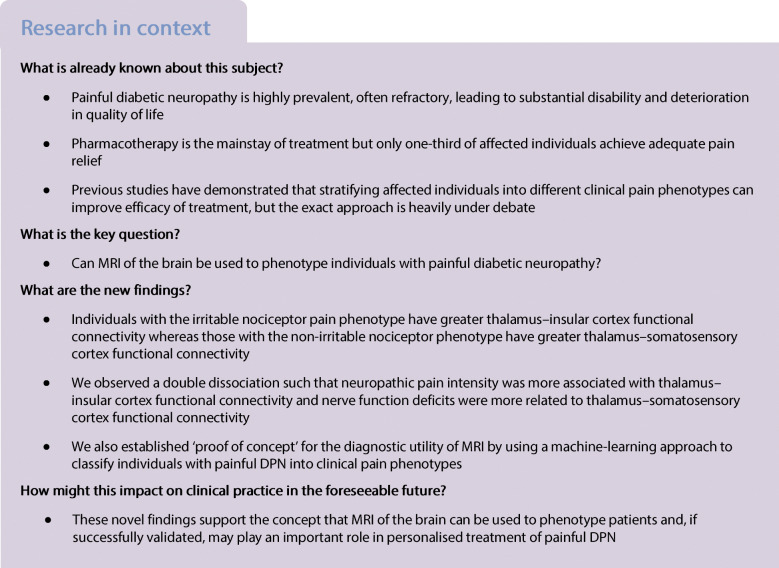



## Introduction

Painful distal symmetrical peripheral neuropathy is highly prevalent in individuals with diabetes and is often refractory, causing substantial disability and deterioration in quality of life. Pharmacotherapy is the mainstay of treatment but the best we can hope for is 50% pain relief in only one-third of patients [1]. This wide variability in treatment response may in part be due to an underlying heterogeneity in clinical pain phenotypes [2]. Using quantitative sensory assessments, individuals with painful diabetic polyneuropathy (DPN) can be broadly subdivided into two phenotypes: irritable (IR), presenting as sensate or relatively preserved sensory function associated with thermal and/or mechanical hyperalgesia; and non-irritable (NIR), presenting as insensate (i.e. dominated by thermal and mechanical sensory loss) [3]. Subsequent studies suggest that some treatments are more effective in patients with the IR compared with the NIR nociceptor phenotype [4]. Consequently, pain phenotyping may become important in guiding individual patients’ treatment, although the exact approach is heavily debated.

Resting-state functional MRI (RS-fMRI) is a quick, non-invasive technique for examining brain function during resting conditions. It utilises spontaneous fluctuations in blood oxygen level-dependent (BOLD) signal to identify brain areas of increased or decreased neuronal activity while the individual lies quietly. The analysis involves identification of correlations in BOLD signal between remote brain areas, referred to as functional connectivity [5]. This recent advance offers huge promise for improving the clinical applicability of functional MRI.

We have previously reported structural and functional central nervous system alterations in individuals with ‘painful hypoaesthesia’ [6] or the painful/painless diabetic foot, which is most closely related to the NIR phenotype. We have also demonstrated how central nervous system changes relate to treatment response in painful DPN [7]. Hence, we hypothesise that in individuals who have painful DPN, alterations in somatosensory network functional connectivity differentiate those with the IR phenotype from those with the NIR phenotype. The primary aim of this study was to examine RS-fMRI functional connectivity in individuals who have painful DPN with the IR and the NIR phenotype. Our secondary aim was to demonstrate ‘proof of concept’ that machine-learning approaches can be used on neuroimaging datasets to classify individuals with painful DPN into sensory phenotypes. If successful, this would provide an alternative, objective, novel method for assessing an individual’s pain phenotype.

## Methods

### Study population

Forty-three, right-handed individuals with painful DPN, aged 18–65 years, with pain duration for at least 6 months, were consecutively recruited from attendees at Sheffield Teaching Hospital NHS Trust painful DPN clinics. Individuals with concurrent severe psychiatric disorders, moderate-to-severe pain from other causes, non-diabetic neuropathies, epilepsy, recurrent severe hypoglycaemia and other factors that would preclude MRI were excluded. The Institutional Review Board of the Sheffield Research Ethics Committee approved the study. All participants provided written informed consent for study participation.

### Definition of painful DPN

Painful DPN was defined as a combination of neuropathic symptoms (Neuropathy Total Symptom Score-6 [NTSS-6]) [8] and signs (Toronto Clinical Neuropathy Score [TCNS] >5) [9], and was confirmed by abnormalities noted in nerve conduction studies (see electronic supplementary material [ESM] Methods) using the American Academy of Neurology and American Association of Electrodiagnostic Medicine recommendations for the minimum case definition criterion for confirmation of DPN [10]. There were 16 sural nerve (2 IR and 14 NIR) and 16 peroneal nerve conduction responses (2 IR and 14 NIR) that were not recordable. The NTSS-6 evaluates the frequency and intensity of individual neuropathy sensory symptoms identified frequently by those with DPN: numbness and/or insensitivity; prickling and/or tingling sensation; burning sensation; aching pain and/or tightness; sharp, shooting, lancinating pain; and allodynia and/or hyperalgesia [8]. The TCNS is a screening tool for DPN and correlates with DPN severity. It uses a simplified neurological examination to assess peripheral sensory perception and the presence of neuropathy symptoms [9].

### Sensory phenotyping and quantitative sensory assessments

Quantitative sensory testing (QST) is a means of assessing sensory phenotype and differences in QST variables may give insight into pathophysiological mechanisms. All participants with painful DPN underwent QST of the feet using the protocol developed by the German Research Network of Neuropathic Pain (DFNS) [11]. GS, MA and FH-G underwent formal training in conducting the DFNS QST protocol at Mannheim University using healthy volunteers. The QST results were used to classify participants into IR and NIR nociceptor phenotypes. Cold and warm detection thresholds, as well as cold and heat pain thresholds and thermal sensory limens (including paradoxical heat sensations), were established using a MEDOC TSA-II Neurosensory Analyser (Ramat Yishai, Israel). We also tested mechanical detection and pain thresholds and mechanical pain sensitivity, allodynia, pressure pain thresholds (PPTs), wind-up ratio (WUR) and vibration detection thresholds. The mechanical detection threshold was assessed with a set of standardised von Frey filaments (0.25, 0.5, 1, 2, 4, 8, 16, 32, 64, 128 and 256 mN; Nervtest, Marstock, Germany) using a modified method of limits. The mechanical pain threshold was assessed with a set of seven metal probes of standardised stimulus intensities (8, 16, 32, 64, 128, 256 and 512 mN; MRC Systems – Medizintechnische Systeme, Heidelberg, Germany), using a uniform skin contact area of 0.25 mm and a modified method of limits. The mechanical pain sensitivity of the skin and dynamic mechanical allodynia were determined using the same set of seven metal probes with standardised stimulus intensities and, in addition, a set of seven light intensity stimuli: a cotton wool ball with a force of 3 mN; a Q-tip (fixed to a plastic stick) with a force of 100 mN; and a paintbrush with an applied force of 200–400 mN. These stimuli were applied 50 times (five runs of ten stimuli per test site in different pseudo-randomised sequence), and the participants were asked to rate the intensity of each stimulus on a 0–100 numeric rating scale (0, no pain; 100, most severe pain). The WUR, as a measure of enhanced temporal summation, was examined by a pinprick stimulus of standardised intensity (256 mN). The stimulus was first applied singularly and then in a series of ten stimuli with a frequency of 1 Hz within an area of 1 cm^2^. Participants were asked to rate the intensity of the first stimulus and the mean of ten stimuli on a scale of 0–100. The ratio between the two measures was calculated as WUR; a WUR of >1 indicates enhanced temporal summation. The vibration detection threshold was examined using a tuning fork (64 Hz, 8/8 scale) at the (lateral or medial) malleolus area. Muscular pressure pain threshold was examined by applying mechanical pressure at a rate of 0.5 kg/s (Algometer, Somedic, Sweden) at the abductor halluces muscle. Except for the vibration detection threshold and pressure pain threshold, all sensory tests were performed in the S1 dermatome bilaterally (unless defined by the distribution of symptoms). Participants were familiarised with the testing procedure on the dorsum of the forearm before all variables were measured over the dorsum of both feet (S1 dermatome). PPTs were recorded over the arch of the foot and vibration detection thresholds were tested over the medial malleolus. The QST data were entered into the data analysis system eQUISTA provided by the DFNS. eQUISTA transformed the raw QST data into *z* scores thus normalising for age, sex and the body location of testing [12]. Positive *z* scores denote gain of function, whereas negative *z* scores denote loss of function. Based on quantitative sensory assessment findings, participants were divided into IR nociceptor phenotype (defined as the presence of either dynamic mechanical allodynia, reduced mechanical or pressure threshold, increased mechanical pain sensitivity, or lower cold or heat pain threshold, or any combination of these signs of hyperexcitability) or NIR nociceptor phenotype (participants not classified as IR nociceptor phenotype [i.e. sensory loss with no signs of hyperexcitability]) using recommendations previously described [13].

### MRI acquisition and analyses

Anatomical data were acquired using a T1-weighted magnetisation prepared rapid acquisition gradient echo sequence (repetition time [TR] 7.2 ms, echo time [TE] 3.2 ms, flip angle 8° and voxel size 0.9 mm^3^, yielding isotropic spatial resolution). A 6 min resting-state fMRI sequence was acquired while participants fixated on a cross using a T2*-weighted pulse sequence (TE 35 ms; TR 2600 ms, in-plane pixel dimensions 1.8 mm × 1.8 mm, contiguous trans-axial slices 4 mm thick). MRI was performed at 3.0 T (Ingenia; Phillips Medical Systems, Best, Holland).

Ten regions of interest (ROIs) involved in somatic and non-somatic pain processing were chosen for analyses: bilateral primary somatosensory cortex (S1), motor cortex (M1), insular cortex, anterior cingulate gyrus and thalamus. RS-fMRI analysis was performed using the NITRC Functional Connectivity (CONN) Toolbox 18.b (www.nitrc.org/projects/conn) [14] and SPM8 (Wellcome Trust Centre for Neuroimaging London, UK) in Matlab 2019a (the MathWorks, Natick, MA, USA). Functional connectivity matrices between the pre-specified ROIs were calculated and the IR vs NIR nociceptor phenotype interaction was examined. The significance of ROI-to-ROI connection was determined through false-positive control false discovery rate (FDR)-corrected *p* values with a χ^2^ test with two-sided inferences [14]. Cortical reconstruction and volumetric segmentation were performed with FreeSurfer software (http://surfer.nmr.mgh.harvard.edu) to obtain anthropometric measures for each of the ROIs. These results were used to adjust for regional morphological differences in the resting-state functional connectivity analyses.

### Machine-learning methods

We classified participants with the IR and NIR nociceptor phenotypes using a hyperparameter tuned support vector machine (SVM) classifier. Of the 55 total participant labels used, 14 participants (0.25 testing set) were used to train and the rest to test our classifier performance. The ten sources chosen a priori were as described in the resting-state processing step. These sources were also the features extracted from the structural and volumetric analysis. Of these, the most relevant features from both the resting-state and the T1 image analysis were chosen using a cross-validated recursive feature elimination method. Lastly, a tenfold cross-validation was implemented to reduce out of sample bias. All our analyses were performed using the Scikit-learn package in Python version-0-22-0 (https://github.com/scikit-learn/scikit-learn) [15]. The performance of the machine-learning algorithm to classify participants with the NIR nociceptor phenotype was determined by the area under the receiver-operating-characteristic curve, accuracy and F1 scores. Our classifier was also optimised and the following hyperparameter tuning values were used in our SVM classifier.

- Regularisation parameter: C = 100 chosen as imbalanced datasets benefits from a higher C value [16]
- L2 penalty: we chose L2 as a conventional approach to regularisation [17]
- Early gradient descent stop at 1 × 10^−4^: this default value was chosen to optimise speed
- No class weights: this is the default parameter
- Radial basis function kernel: this was easy to calibrate, is a non-parametric model enabling better model selection, and has been shown to perform better than linear and polynomial kernels [18].

### Statistical analysis

A *p* value of  < 0.05 was considered statistically significant. Categorical variables were expressed as numbers and percentages and were compared using Fisher’s exact or ordinal χ^2^ tests as appropriate. Continuous variables were expressed as medians and IQRs or as means and SDs, as appropriate, and were compared using Student’s *t* tests (SPSS Statistics for Windows, version 26.0; IBM, Armonk, USA). In addition, *z* values of functional connectivity that were significantly correlated to severity of neuropathy (TCNS) and pain scores (NTSS-6) were determined using Pearson correlation for normally distributed data and Spearman Rank correlation for non-normally distributed data. The *z* score was chosen as it is assumed to be more appropriate than the magnitude of difference because it also considers the variance in the signal. Finally, we statistically compared the partial correlation coefficients using the Steiger *z* transform test [19] implemented on the web version (https://blogs.gwu.edu/weissba/teaching/calculators/hotellings-t-and-steigers-z-tests/).

## Results

Forty-three participants (10 IR nociceptor phenotype, 33 NIR nociceptor phenotype) completed the study. There was no significant difference in age, duration or type of diabetes, or duration of pain or type of pain medication between study groups (Table 1). Participants with the IR nociceptor phenotype (vs NIR nociceptor phenotype) had reduced cold pain threshold (*p*=0.05) and greater mechanical pain sensitivity and PPT (*p* < 0.05, ESM Fig. 1). A significantly higher proportion of participants with the IR nociceptor phenotype displayed dynamic mechanical allodynia (χ^2^ 26.0; *p* < 0.001) and paradoxical heat sensation (χ^2^ 10.9; *p* = 0.001) when compared with participants with the NIR nociceptor phenotype.

**Table 1:** Clinical and neurophysiological characteristics of the study participants

Characteristic	IR nociceptor phenotype	NIR nociceptor phenotype	*p* value
*N*	10	33	
Age, years	56.9 (12.9)	58.4 (11.2)	0.74
Male sex, *n* (%)	9 (90.0)	20 (60.6)	0.08
Type of diabetes, *n* type 1/*n* type 2	3/7	10/23	0.65
Duration of diabetes, years	17.2 (9.5)	18.4 (13.1)	0.78
Duration of pain, years	8.9 (5.6)	8.3 (7.0)	0.80
HbA_1c_, mmol/mol	69.1 (17.6)	67.6 (15.0)	0.79
HbA_1c_, %	8.5 (3.8)	8.3 (3.5)	
NTSS-6 score	16.5 (3.0)	13.1 (5.0)	0.02
TCNS	19.7 (4.6)	16.1 (9.0)	0.26
Medications, *n* (%)			
Pregabalin/gabapentin	7 (70.0)	14 (42.4)	0.12
Duloxetine	6 (60.0)	10 (30.3)	0.14
Amitriptyline	0 (0)	5 (15.2)	0.32
Opiates	5 (50.0)	11 (33.3)	0.46
Other	0 (0)	2 (6.1)	0.59
Sural nerve^a^			
Conduction velocity, m/s	34.2 (9.7)	39.0 (8.1)	0.30
Amplitude, mAmp	3.8 (5.7)	2.9 (6.7)	0.73
Common peroneal nerve^b^			
Conduction velocity, m/s	37.6 (5.0)	35.4 (6.1)	0.41
Amplitude, mAmp	4.7 (2.0)	4.4 (4.2)	0.87
Distal latency (ms)	3.6 (2.4)	1.4 (1.3)	0.007
DFNS QST (*z* scores)			
Cold detection threshold	−2.64 (0.6)	−2.24 (1.1)	0.28
Warm detection threshold	−1.83 (0.3)	−1.87 (0.5)	0.79
Thermal sensory limens	−2.20 (0.6)	−2.13 (0.7)	0.80
Cold pain threshold	−1.00 (0.1)	−0.73 (0.6)	0.05
Heat pain threshold	−1.46 (0.3)	−1.38 (0.5)	0.61
PPT	1.80 (1.6)	−0.83 (2.2)	0.05
Mechanical pain threshold	−1.43 (1.6)	−1.71 (1.6)	0.65
Mechanical pain sensitivity	1.07 (2.1)	−1.00 (1.6)	0.003
WUR	1.03 (2.2)	0.03 (1.3)	0.12
Mechanical detection threshold	−3.35 (1.3)	−3.20 (1.6)	0.92
Vibration detection threshold	−2.66 (2.4)	−3.41 (2.3)	0.39
Brain morphometry			
Somatosensory cortex			
Surface area, mm^2^	578.2 (64.3)	535.3 (54.1)	0.04
Vertices^c^	988.7 (96.1)	913.4 (115.1)	0.05
Volume, mm^3^	1481.9 (186.2)	1424.7 (185.8)	0.39
Thalamus			
Right volume, mm^3^	6475.0 (701.3)	5874.4 (626.3)	0.01
Left volume, mm^3^	7327.0 (894.6)	7039.5 (1145.7)	0.47
Anterior cingulate cortex			
Thickness, mm	2.38 (0.2)	2.57 (0.2)	0.02
Volume, mm^3^	1773.5 (390.1)	1773.5 (321.7)	0.99
Vertices^c^	1032.1 (236.1)	982.7 (165.2)	0.46
Motor cortex			
Surface area, mm^2^	4719.8 (606.6)	4535.9 (413.6)	0.28
Vertices^c^	7347.2 (935.5)	7094.8 (746.0)	0.38
Volume	11,562.3 (1410.4)	11,167.7 (1218.5)	0.39
Insular cortex			
Thickness, mm	2.87 (0.2)	2.80 (0.1)	0.27
Volume, mm^3^	6056.6 (965.9)	6016.3 (677.7)	0.88
Vertices^c^	3167.3 (428.1)	3182.6 (357.5)	0.91

Data are shown as mean (SD), except where they are reported as *n* (%)

^a^Twenty-three (NIR 19, IR 4) sural nerve conduction responses were not recordable

^b^Twelve (NIR 11, IR 1) peroneal nerve conduction responses were not recordable

^c^Structural measure vertices are expressed as an arbitrary unit of measurement

**Fig. 1:**
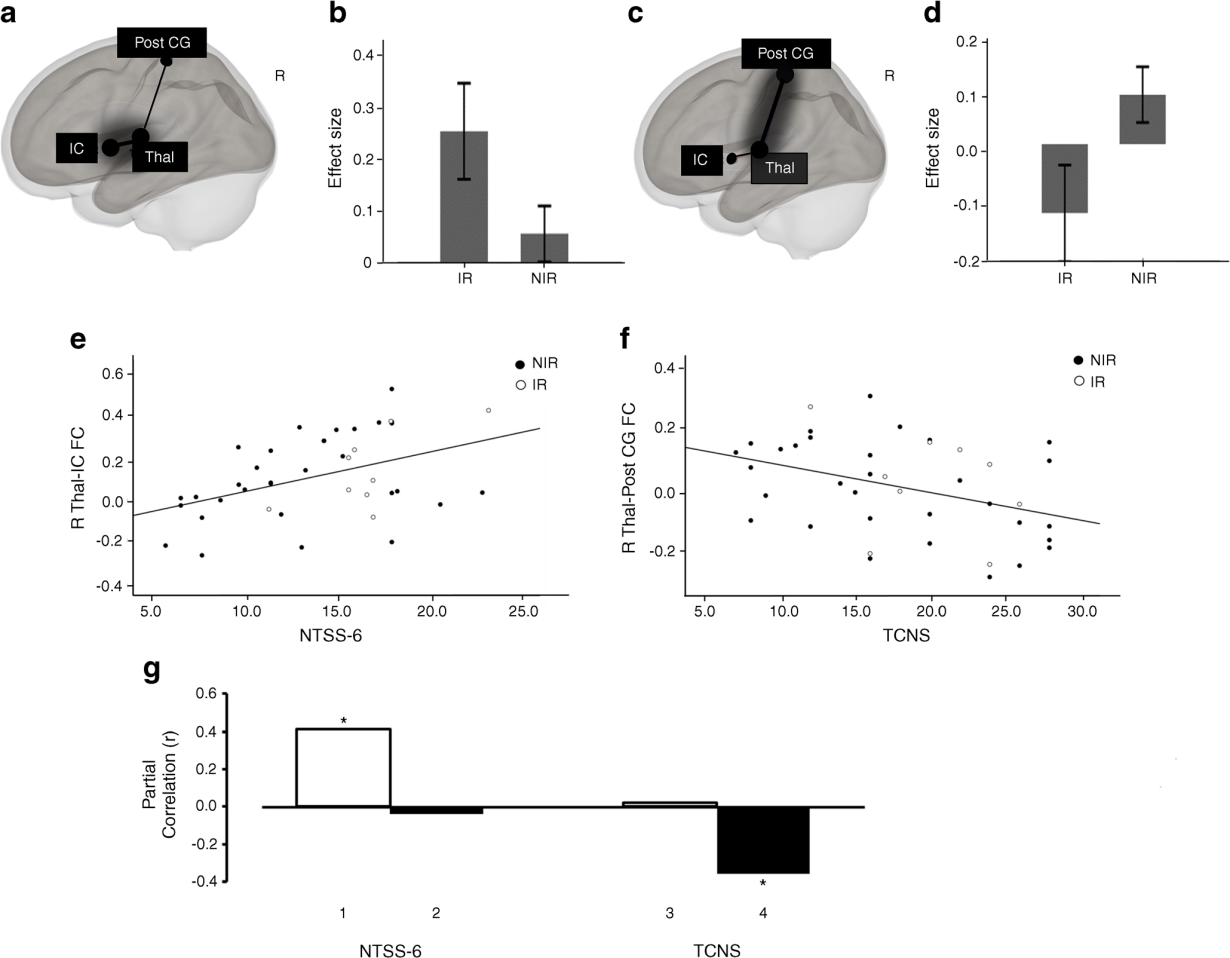
(**a**–**d**) Right view of resting-state functional connectivity in individuals with painful DPN who had the IR nociceptor phenotype (**a**) and NIR nociceptor phenotype (**c**); R, right; IC, insular cortex (Montreal Neurological Institute [MNI] coordinates: 44, 4, 0); Post CG, postcentral gyrus (MNI coordinates: 2, −36, 62); Thal, thalamus (MNI coordinates: 10, −19, 6). Bar charts show the effect size of differences in mean thalamus–insular cortex (**b**) and thalamus–postcentral cortex (**d**) functional connectivity between study groups (error bars represent 95% CI). (**e**, **f**) Scatter-plots depicting linear correlation between the right thalamus–insular cortex functional connectivity (R Thal-IC FC) and the NTSS-6 pain scores (**e**) and between the right thalamus–somatosensory cortex functional connectivity (R Thal-Post CG FC) and the TCNS (**f**). (**g**) Bar graph plotting four functional connectivity and behaviour partial correlation coefficients (Pearson’s *r*) derived from the functional connectivity of the right thalamus–insular cortex (R Thal-IC FC, white bars) and the right thalamus–somatosensory cortex (R Thal-Post CG FC, black bars). Bars 1 and 2 indicate correlations involving the NTSS-6 and bars 3 and 4 indicate correlations involving the TCNS. Each partial *r* (e.g. bar 1) is obtained by correlating a given behaviour (e.g. self-reported pain scores, NTSS-6) with the functional connectivity derived from a given network (e.g. right Thal-IC FC). *p<0.05

RS-fMRI data was unavailable for three participants (IR 1, NIR 2). In participants with the IR nociceptor phenotype, there was significantly greater resting functional connectivity between the thalamus and insular cortex (Fig. 1b; β = 0.2, T(38) = 3.11; *p*-FDR = 0.03) when compared with participants with the NIR nociceptor phenotype. Conversely, there was an opposing pattern for thalamus–somatosensory cortex functional connectivity; participants with the IR nociceptor phenotype displayed decreased functional connectivity compared with those having the NIR nociceptor phenotype (Fig. 1d; β = −0.22, T(38) = −4.98, *p*-FDR = 0.03). There were no significant group differences in functional connectivity between the other ROIs examined.

Given the relatively low correlation between self-reported pain and neuropathy severity scores (*r* = −0.03, *p* = 0.85), we investigated whether these measures were associated with different patterns of thalamic resting-state functional connectivity. Specifically, we tested the double dissociation such that NTSS-6 pain scores were more associated with thalamus–insular cortex functional connectivity, while the TCNS scores were more associated with thalamus–somatosensory cortex functional connectivity. We observed two significant partial correlations: one linking NTSS-6 pain scores to thalamus–insular cortex functional connectivity (Fig. 1e,g, bar 1; *r* = 0.41; *p* = 0.01) and the other linking TCNS to thalamus–somatosensory cortex functional connectivity (Fig. 1f,g, bar 4; *r* = −0.35; *p* = 0.03). These results indicate that individuals with greater thalamus–insular cortex functional connectivity exhibit higher self-reported pain scores (in keeping with the IR nociceptor phenotype group having significantly greater NTSS-6 score than the NIR phenotype group; Table 1, *p* = 0.02), while individuals with lower functional connectivity of the thalamus–somatosensory cortex have a larger neuropathy deficit. By contrast, the partial correlations between NTSS-6 and thalamus–somatosensory cortex functional connectivity (Fig. 1g, bar 2) and between TCNS and thalamus–insular cortex functional connectivity (Fig. 1g, bar 3) were not significant. Of note, the Steiger *z* transform test revealed that the magnitude of the partial *r* encompassing the NTSS-6 score was significantly greater for thalamus–insula cortex than for thalamus–somatosensory cortex functional connectivity (Fig. 1g, bar 1 vs bar 2; two-tailed Steiger *z* test, *z* = 2.07, *p* = 0.04). By contrast, the magnitude of the partial *r* encompassing the TCNS score was significantly larger for thalamus–somatosensory cortex than for thalamus–insula cortex (Fig. 1g, bar 3 vs bar 4; two-tailed Steiger *z* test, *z* = 2.02, *p* = 0.04). These analyses revealed a double dissociation connecting self-reported pain scores selectively and preferentially to the functional connectivity of the thalamus–insular cortex within the right hemisphere. By contrast, neuropathy deficit was related to the reduction in the functional connectivity of the thalamus–somatosensory cortex.

Next, we examined whether the functional connectivity–neuropathy scores dissociation was driven by structural changes in the thalamus, insular cortex or somatosensory cortex (Table 1). We repeated the partial correlation analysis but we also regressed out the volumes measurements of each of these brain regions. Once again, the partial correlations linking self-reported pain scores (NTSS-6) to the thalamus–insular cortex functional connectivity (*r* = 0.37, *p* = 0.03) and linking neuropathy severity (TCNS) to thalamus–somatosensory cortex functional connectivity (*r* = 0.35, *p* = 0.03) were demonstrated. Hence, structural changes to the thalamus, insular cortex and somatosensory cortex do not account for the functional connectivity–neuropathy score double dissociation.

Finally, group classification performance of the machine-learning model for the NIR phenotype achieved an accuracy of 0.92 (95% CI 0.08) and sensitivity of 90%. The positive predictive value (NIR) and negative predictive value (IR) was 100% and 67%, respectively. AUC analysis indicated that the machine-learning model exhibited good performance accuracy.

## Discussion

The key findings from this study were that individuals with the IR nociceptor phenotype have significantly greater thalamus–insular cortex functional connectivity and decreased thalamus–somatosensory cortex functional connectivity compared with those with the NIR nociceptor phenotype. Indeed, there was a significant positive correlation between thalamus–insular cortex functional connectivity and pain scores (NTSS-6). Similar associations between insular cortex functional connectivity and pain scores have also been demonstrated across other chronic pain conditions [20]. Thus, the insular cortex, which plays a pivotal role in affective and attentional pain processing [21], may be an overactive pain-promoting brain region in individuals with the IR nociceptor phenotype. In addition, there was a greater reduction in thalamus–somatosensory cortex functional connectivity in individuals with more severe neuropathy (TCNS). This suggests that the deafferentation or dying-back axonopathy, resulting from a severe neuropathy, lead to reduced peripheral sensory input which in turn leads to a reduction in somatosensory cortical volume [6] and functional connectivity. Crucially, we observed a double disassociation such that deficits of nerve function were more correlated with thalamus–somatosensory cortex functional connectivity and self-reported pain scores were more correlated with thalamus–insular cortex functional connectivity. Taken together, we have demonstrated how MRI measures of functional connectivity relate to both the somatic (i.e. TCNS) and non-somatic (self-reported pain intensity ratings) assessment of painful DPN. To the best of our knowledge, this is the first time this has been demonstrated in painful DPN. Future prospective studies are required to determine the natural history of the alterations in functional connectivity described in relation to the onset of painful DPN.

Current therapies for painful DPN have limited efficacy, as reflected in the high psychosocial burden, low rates of functional recovery and return to work, and continued reliance on opioid analgesics [1, 22, 23]. The lack of a reliable biomarker to stratify patients and to predict therapeutic response is one of the main barriers preventing the identification and development of safe and effective, non-additive pain medications. We have demonstrated how structural and functional changes within the central nervous system reflect an individual’s clinical pain phenotype [6] and also predict response to neuropathic pain treatment [7]. There is now increasing evidence that magnetic resonance neuroimaging could serve as a reliable biomarker in clinical trials of pain therapeutics. This could increase the probability of novel compounds advancing to Phase II trials, reduce the variability of the therapeutic response and reduce the overall expense and time of drug development.

We used machine-learning approaches to integrate MRI anatomical data and resting-state functional connectivity data to classify individuals with painful DPN into sensory phenotypes. We found this approach is feasible with a good degree of accuracy and performance. Although these findings are promising, more research is now needed to externally validate our machine-learning model with a larger sample size. If successfully validated, multidimensional reduction of magnetic resonance neuroimaging data through machine-learning offers a novel approach to classify patients with painful DPN into sensory phenotypes.

This study has some limitations, including a study population with a male predominance and a long pain duration. To assess this further, we completed a sensitivity analysis by examining differences in functional connectivity between sexes (male vs female) and between individuals with long (>8 years) vs short (<8 years) duration of pain (mean duration of pain in the whole study population was 8.5 [SD 6.9] years). We found no significant differences in functional connectivity in these two comparisons. Nevertheless, further studies are required to determine whether the findings in this study are reproducible across sexes and individuals with different pain durations. Another limitation was the lack of a painless DPN control group. This would have enabled us to determine the differential impact of neuropathy vs the presence of pain. However, our study findings provide clues to address this limitation. There was a clear disassociation between deficits of nerve function that correlated with thalamus–somatosensory cortex functional connectivity and self-reported pain scores, which were more correlated with the thalamus–insular cortex functional connectivity. Future studies should consider the inclusion of individuals with painless DPN to explore this further.

In summary, there has been considerable progress towards a mechanism-based approach to managing painful DPN in recent years [24]. Much of the focus has been on sensory profiling using QST, which remains a subjective psychophysical measure. Crucially, these methods do not capture the complex multifaceted experience of pain, which not only affects sensory but also emotional/cognitive processing. What is clear is that many of the factors influencing pain perception are centrally mediated and neuroimaging provides the best tool to quantify this. Using advanced multimodal magnetic resonance neuroimaging, we have demonstrated alterations in pain-processing brain regions that relate to clinical pain phenotype, treatment response [7] and behavioural/psychological factors impacted by pain [6]. Taken together, these assessments could serve as a possible central pain signature for painful DPN. The challenge now, is to apply this potential pain biomarker at an individual level in order to demonstrate clinical utility. To this end, we have shown proof of concept that a machine-learning approach to classify individuals into different clinical pain phenotypes using brain imaging features taken from a quick, 6 min RS-fMRI scan is feasible. In future studies we aim to externally validate and optimise this model on a larger cohort of individuals and examine whether and how such a model can be used as a biomarker in clinical trials of pain therapeutics.

## Supplementary information

ESM (PDF 73.0 kb)

## Data Availability

The datasets generated and/or analysed during the current study are available from the corresponding author on reasonable request.

## Abbreviations

BOLD: Blood oxygen level-dependent
DFNS: German Research Network of Neuropathic Pain
DPN: Diabetic polyneuropathy
FDR: False discovery rate
NTSS-6: Neuropathy Total Symptom Score-6
PPT: Pressure pain threshold
QST: Quantitative sensory testing
ROI: Regions of interest
RS-fMRI: Resting-state functional MRI
SVM: Support vector machine
TCNS: Toronto Clinical Neuropathy Score
TE: Echo time
TR: Repetition time
WUR: Wind-up ratio
