# Supplementary material for: Somatosensory network functional connectivity differentiates clinical pain phenotypes in diabetic neuropathy
Source: Diabetologia. 2021 Mar 25;64(6):1412–21. doi: 10.1007/s00125-021-05416-4 (PMC8099810; doi:10.1007/s00125-021-05416-4)
Supplement: Supplementary file 1 — (PDF 73.0 kb) [file 125_2021_5416_MOESM1_ESM.pdf]

Electronic Supplementary Materials

ESM Methods: Nerve Conduction Studies

The American Academy of Neurology and American Association of Electrodiagnostic Medicine recommendations was used to confirm the presence of DPN. The minimum case definition criteria used was an abnormality of any attribute of nerve conduction in 2 separate nerves, one of which was the sural nerve [3]. Nerve conduction studies were performed at a stable skin temperature of 31°C and a room temperature of 24°C using a Medelec electrophysiological system using surface electrodes (Synergy Oxford Instruments, Oxford, U.K.). A warm water bath was used to warm cool limbs prior to testing and maintained, if necessary, using an external electric heater. The limb temperature refers to surface temperature at the point of assessment using a contact temperature probe. The following nerve attributes were measured: a) sural sensory nerve action potentials and conduction velocities and b) common peroneal and tibial motor nerve distal latency, compound muscle action potential, and conduction velocity. Variables such as age, height, sex and weight were measured and accounted for when interpreting nerve conduction results.

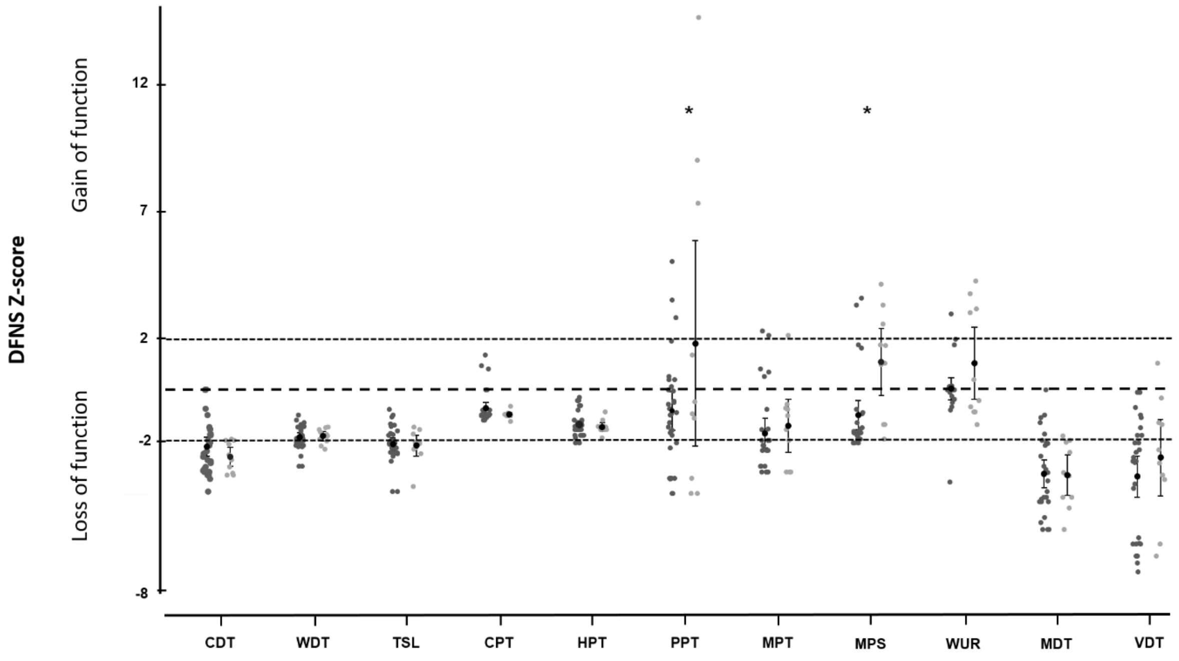

ESM Figure 1: Scatter plot and mean ± 95%CI of z-scores for quantitative sensory testing (QST) parameters in study patients with painful diabetic neuropathy with the irritable nociceptor (IR) and non-irritable nociceptor phenotype. DFNS, German Research Network on Neuropathic pain. * $p<0.05$, Student t-test. CDT, cold detection threshold; CPT, cold pain threshold; HPT, heat pain threshold; MDT, mechanical detection threshold; MPS, mechanical pain sensitivity; MPT, mechanical pain threshold; PPT, pressure pain threshold; TSL, thermal sensory limen; VDT, vibration detection threshold; WDT, warm detection threshold; WUR, wind-up ratio. Dark and light circles represent non-irritable and irritable nociceptor phenotypes respectively.

ESM References

1. England JD, Gronseth GS, Franklin G, Miller RG, Asbury AK, Carter GT et al. Distal symmetric polyneuropathy: a definition for clinical research: report of the American Academy of Neurology, the American Association of Electrodiagnostic Medicine, and the American Academy of Physical Medicine and Rehabilitation. Neurology 2005; 64:199-207.
